# Recognizing Healthcare Needs Among Older Adults With Dementia and With Migrant Background

**DOI:** 10.1093/geroni/igaa057.535

**Published:** 2020-12-16

**Authors:** Mary Dioise Ramos

**Affiliations:** Kennesaw State University, Marietta, Georgia, United States

## Abstract

Dementia, a syndrome affecting millions of people worldwide, can be challenging, especially in patients with a migrant background. Language barriers and language-based diagnostic tools, cultural differences in the perception of the syndrome as well as restricted access to healthcare can influence medical care. Healthcare providers play a key role in diagnosing persons with dementia and they are in the best position to raise awareness about dementia among older adults. They examine a large number of patients and are generally the first point of contact for people with any health complaints. Therefore, they are able to identify treatable causes of the syndrome at an early stage to prevent irreversible health impairment. This study investigates whether health care providers feel prepared to meet the diagnostic needs of these patient groups and whether there are challenges and support needs. A cross-sectional study is used in this study among a random sample of healthcare providers. Healthcare providers experienced barriers especially the uncertainties in identifying dementia in patients with a migrant background. Language barriers that affected or prevented diagnostics and information deficits in patients with a migrant background were the most frequently reported barriers. Lack of acceptance of the syndrome was also common. Public health measures supporting healthcare providers in their interaction with a migrant background patient as well as information and services for patients with dementia are needed. Efforts to facilitate access to interpreting services and to focus on people with a migrant background in healthcare are necessary.

